# Revisiting the identification of *Syllipsimopodi bideni* and timing of the decabrachian-octobrachian divergence

**DOI:** 10.1038/s41467-023-42842-x

**Published:** 2023-12-07

**Authors:** Christian Klug, Kevin Stevens, René Hoffmann, Michał Zatoń, Thomas Clements, Martin Košťák, Robert Weis, Kenneth De Baets, Jens Lehmann, Jakob Vinther, Dirk Fuchs

**Affiliations:** 1 Paläontologisches Institut und Museum, Universität Zürich, Zürich, Switzerland; 2 Institute of Geology, Mineralogy and Geophysics, Ruhr University Bochum, Bochum, Germany; 3 Institute of Earth Sciences, University of Silesia in Katowice, Sosnowiec, Poland; 4 GeoZentrum Nordbayern, Department of Geography and Geosciences, Friedrich-Alexander Universität Erlangen-Nürnberg, Erlangen, Germany; 5 Institute of Geology and Palaeontology, Faculty of Science, Charles University, Prague 2, Czech Republic; 6 Musée national d’histoire naturelle, rue Münster, Luxembourg; 7 Institute of Evolutionary Biology, Faculty of Biology, Biological and Chemical Research Centre, University of Warsaw, Warsaw, Poland; 8 Fachbereich Geowissenschaften, Universität Bremen, Bremen, Germany; 9 Schools of Earth Sciences and Biological Sciences, University of Bristol, Bristol, UK; 10 Bayerische Staatssammlung für Paläontologie und Geologie, München, Germany

**Keywords:** Palaeontology, Taxonomy, Phylogenetics

**arising from** C.D. Whalen & N.H. Landman. *Nature Communications* 10.1038/s41467-022-28333-5 (2022)

Recently, on the basis of a single specimen (ROMIP 64897) from the Royal Ontario Museum (Canada), Whalen and Landman^1^ described the new coleoid taxon with a fairly completely preserved frontal part as *Syllipsimopodi bideni*. The specimen, recovered from the Bear Gulch Limestone, Heath Formation in Fergus County, Montana, USA, is of Serpukhovian age^2^. Based on the suggested presence of a gladius, ten arms, and fins, as well as the supposed absence of a phragmocone, the authors interpreted the “remarkably well-preserved” specimen as “the oldest definitive vampyropod and crown coleoid”^1^. We herein test if the fidelity of preserved characters in *S. bideni* affects the interpretation of this organism—particularly by comparing these characters to other soft bodied cephalopod fossils from Bear Gulch. We provide evidence for the likely synonymy of *S. bideni* and *Gordoniconus beargulchensis*. Our interpretation casts doubt on the phylogeny proposed by Whalen and Landman^1^, who suggested *S. bideni* as the oldest vamyropod. Vampyropoda ( = Octobrachia or Octopodiformes) is considered to be the sister group of all ten-armed cephalopods (Decabrachia)^3–5^, which is also supported by recent molecular analyses^6–8^.

The holotypes of the early coleoids *Gordoniconus beargulchensis*^9^ and *Syllipsimopodi bideni* share many important morphological characters. They are both of the same age and come from the same locality. In our Fig. 1, we show the photographs of both holotypes (Fig. 1a, e) and line drawings made after these published images (Fig. 1b, d) at the same scale. In Fig. 1c, we overlaid the line drawings of both holotypes with the drawing of *G. beargulchensis* being scaled down by 20% to fit the body chamber width to each other. This overlay demonstrates that the morphology and proportions of the preserved parts of the two holotypes are so similar that we consider *S. bideni* may be a subjective junior synonym of *G. beargulchensis*. Importantly, the distinctly tapering body in *S. bideni* with straight margins is identical with the body-chamber portion of *G. beargulchensis*. The few differences can be explained by taphonomic alteration, such as the median ridge in *S. bideni* being the result of compactional fracturing of the mineralised body chamber. The absence of the chambered phragmocone is due to oblique splitting of the rock and without the missing counterpart it is impossible to state definitively that this character is truly absent. Accordingly, the extension of the phylogenetic split of the Decabrachia and Octobrachia (‘Vampyropoda’^1^) into the Early Carboniferous is not supported. Instead, we think that the phylogenies presented earlier^3–5^ as well as recent molecular analyses^6–8^ support a timing of this divergence during the Middle Permian to Early Triassic. The latter theory is based on palaeontological and neontological morphological data, as well as on molecular data derived from modern coleoids; it has been tested repeatedly and found support from several independent lines of reasoning. Furthermore, the supposed Carboniferous octobrachian *Pohlsepia*^10–14^ is a highly doubtful taxon -as stated by Whalen and Landman^1^- and should, therefore, not be used to support the phylogenetic split of the Decabrachia and Octobrachia (‘Vampyropoda’^1^) during the Early Carboniferous.

**Fig. 1:**
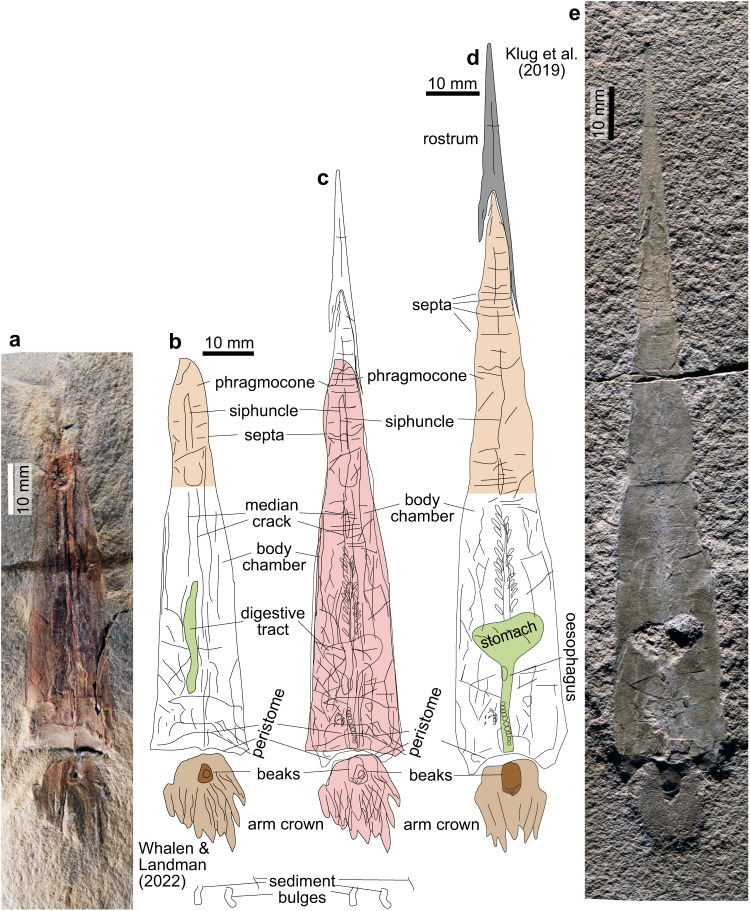
The early coleoids *Gordoniconus beargulchensis* and *Syllipsimopodi bideni* from the Bear Gulch Limestone, Heath Fm., Serpukhovian, Fergus County, Montana, USA. Light brown—phragmocone; middle brown—arm crown; dark brown—buccal mass; green—digestive tract; middle grey—rostrum. **a** Photo of the holotype of *S. bideni* (increased contrast after^1^: suppl. fig. 6), ROMIP 64897. **b** Line drawing after **a**. **c** Overlay of **b** (filled in pink) and **d** (scaled down by about 20% to fit body chamber width). **d** Line drawing after **e**. **e** Holotype of *G. beargulchensis*, AMNH 50267; photo modified after^10^ **b**.

We suggest that the specimen described as *Syllipsimopodi bideni* is synonymous with *Gordoniconus beargulchensis*. They have the same geographic and stratigraphic origin. They share their absolute size (body chamber width differs by 20%, possibly representing slightly different ontogenetic stages or different degrees of compaction), the conch shape (orthoconic conch with an acute apical angle of less than 13°, and a rostrum with an apical angle of ca. 10°), the body chamber shape (width to length ratio, angle of the sides/apical angle, slight terminal apertural constriction), the shallow ventral hyponomic sinus, the broadly rounded dorsal projection, and the narrow mid-ventral siphuncle (interpreted as fin support by^1^). The phragmocone, fragmentarily preserved in the specimen described in^1^, is only slightly longer than the body chamber and has closely spaced, simply dome-shaped septa. The head of both specimens carries an arm-crown with ten moderately strong and rather short arms of similar length, possibly with small circular suckers^1^, probably in double rows per arm. Tentacles are absent. The oesophagus extends about 30 to 50% of the body chamber length. An ink sac is unknown; the position and proportions of the buccal mass and beak remains, the length and shape of the oesophagus (interpreted as ink sac by^1^) and the overall mode of preservation are nearly identical.

The holotypes of *G. beargulchensis* and *S. bideni* are preserved with slightly darker colour of the arm crown, present as a very shallow imprint (due to being entirely soft bodied), the completely demineralized hard parts, the orientation and approximate number of fractures in the shell of the body chamber (including those running parallel to the plain of symmetry), and the phosphatic remains on the phragmocone (supplementary figs. 1–3 in refs. ^15,16^; “presumed connective tissues” *sensu* supplementary figs. 4, 7 in ref. ^1^). The longitudinal structure along the midline in *S. bideni* is similarly preserved in some specimens of *G. beargulchensis* (supplementary fig. 1 in ref. ^9^) and is here interpreted as a median crack formed by compaction. These factors lead us to consider the hypothesis as the most parsimonious that the new specimen^1^ was identified incorrectly as a new taxon, because of the largely missing phragmocone and rostrum; however, it is likely that these parts were lost, probably during extraction (attached to the counterslab?). Further, there is a possibility that these body parts are absent due to taphonomic reasons; dissolution of the aragonitic phragmocone might seem odd but has been documented for ammonoids while organic remains are preferentially preserved in the same specimens from the same locality. Whalen and Landman^1^ also argued that two of the arms might be elongated in their specimen but could not exclude this was a taphonomic artefact; moreover, they did not provide any direct evidence for the hypotheses that these are indeed parts of the animal. They did not find support for other differences between the elongated and shorter arms so either these structures are random folds in the sediment (no evidence for organic remains) or a taphonomic imprint (such as, e.g., a landing mark^10^). It is important to note that the currently oldest fossil evidence for forms with specialised arms derives from the Toarcian stage of the Lower Jurassic^17^.

Another issue with the interpretation of *S. bideni* is that Whalen & Landman^1^ did not explain the peculiar position of what they interpreted as “terminal fin support”. The presence of unpaired fin cartilages enveloping the gladius apex (as suggested by these authors) is unknown in octobrachians. All Mesozoic gladius-bearing octobrachians as well as extant *Vampyroteuthis* are characterized by paired fin cartilages, which are located on both sides of the median field. An apical position behind the conus is only known from few modern squids, namely highly adapted fast swimmers of the decabrachian order Oegopsida. The assumption of an apical unpaired fin cartilage is at odds with our current knowledge about the evolution of fin cartilage and also cephalopod locomotion^15^.

There are several outstanding issues indicating that *S.bideni* is a subjective junior synonym of *Gordoniconus beargulchensis*. Accordingly, we doubt that the morphological characters present in this fossil are sufficient evidence to accurately upend the well-established phylogenetic hypothesis placing the divergence of Octobrachia and Decabrachia between the Middle Permian and the Late Triassic, as it is also corroborated by molecular clock studies^3,6^. We highlight the need to exert caution when analysing soft bodied fossils as taphonomic factors may result in anatomical variation due to decay and timing of preservation. There is a danger of misinterpreting fragmentary, singular specimens lacking a counterpart without proper comparative anatomical and taphonomic analyses.

## Methods

We compared the holotypes of the two coleoids by tracing visible structures in published photos. First, we gently enhanced the contrast using the raster graphics editor Adobe PhotoShop CS6. We saved the photos as single-layer tif-files and imported them into the vector graphic software CorelDraw X8. There, we traced all visible structures independent of their meaning. Subsequently, we homologized those structures displaying sufficient detail. Finally, we overlaid those drawings by adapting their scales to each other to demonstrate the great degree of morphological similarity.

### Reporting summary

Further information on research design is available in the Nature Portfolio Reporting Summary linked to this article.

### Supplementary information

Reporting Summary

## Data Availability

This study is based on published specimens and photos thereof^1,9^. All data come from these two publications^1,9^. The illustrated specimens are stored at the Royal Ontario Museum (Toronto, Canada) and at the American Museum of Natural History (New York, USA).
