# Supplementary material for: Revisiting the identification of Syllipsimopodi bideni and timing of the decabrachian-octobrachian divergence
Source: Nat Commun. 2023 Dec 7;14:8094. doi: 10.1038/s41467-023-42842-x (PMC10703834; doi:10.1038/s41467-023-42842-x)
Supplement: Supplementary file 1 — Reporting Summary [file 41467_2023_42842_MOESM1_ESM.pdf]

nature portfolio

Corresponding author(s): Christian Klug

Last updated by author(s): 2023-07-04

Reporting Summary

Nature Portfolio wishes to improve the reproducibility of the work that we publish. This form provides structure for consistency and transparency in reporting. For further information on Nature Portfolio policies, see our Editorial Policies and the Editorial Policy Checklist.

Please do not complete any field with "not applicable" or n/a. Refer to the help text for what text to use if an item is not relevant to your study.
For final submission: please carefully check your responses for accuracy; you will not be able to make changes later.

Statistics

For all statistical analyses, confirm that the following items are present in the figure legend, table legend, main text, or Methods section.

n/a	Confirmed	
☒	☐	The exact sample size (*n*) for each experimental group/condition, given as a discrete number and unit of measurement
☒	☐	A statement on whether measurements were taken from distinct samples or whether the same sample was measured repeatedly
☒	☐	The statistical test(s) used AND whether they are one- or two-sided<br>*Only common tests should be described solely by name; describe more complex techniques in the Methods section.*
☒	☐	A description of all covariates tested
☒	☐	A description of any assumptions or corrections, such as tests of normality and adjustment for multiple comparisons
☒	☐	A full description of the statistical parameters including central tendency (e.g. means) or other basic estimates (e.g. regression coefficient) AND variation (e.g. standard deviation) or associated estimates of uncertainty (e.g. confidence intervals)
☒	☐	For null hypothesis testing, the test statistic (e.g. *F*, *t*, *r*) with confidence intervals, effect sizes, degrees of freedom and *P* value noted<br>*Give P values as exact values whenever suitable.*
☒	☐	For Bayesian analysis, information on the choice of priors and Markov chain Monte Carlo settings
☒	☐	For hierarchical and complex designs, identification of the appropriate level for tests and full reporting of outcomes
☒	☐	Estimates of effect sizes (e.g. Cohen's *d*, Pearson's *r*), indicating how they were calculated

Our web collection on statistics for biologists contains articles on many of the points above.

Software and code

Policy information about availability of computer code

Data collection	All data come from the literature, cited in the paper.
Data analysis	Does not apply.

For manuscripts utilizing custom algorithms or software that are central to the research but not yet described in published literature, software must be made available to editors and reviewers. We strongly encourage code deposition in a community repository (e.g. GitHub). See the Nature Portfolio guidelines for submitting code & software for further information.

Data

Policy information about availability of data

All manuscripts must include a data availability statement. This statement should provide the following information, where applicable:

- Accession codes, unique identifiers, or web links for publicly available datasets
- A description of any restrictions on data availability
- For clinical datasets or third party data, please ensure that the statement adheres to our policy

All data used is fully available freely since it is already published. The papers are cited in our manuscript.

Field-specific reporting

Please select the one below that is the best fit for your research. If you are not sure, read the appropriate sections before making your selection.

☐ Life sciences ☐ Behavioural & social sciences ☒ Ecological, evolutionary & environmental sciences

Ecological, evolutionary & environmental sciences study design

All studies must disclose on these points even when the disclosure is negative.

Study description	We discuss a fossil presented in the paper by Whalen & Landman (2022). We compare it to the holotype of Gordoniconus, which has been described repeatedly.
Research sample	We compare the holotypes of S. bideni and G. beargulchensis. We studied the type material of the latter & several further specimens, which were published by Klug et al. (2019; Comms. Biol.). These materials are readily available in the reporitories stated in the manuscript.
Sampling strategy	Since we discuss these two species and S. bideni is known from only one specimen, we basically studied all the reasonably preserved material that is available. This species is not very common.
Data collection	Data collection is limited to a few measurements taken from the two holotypes. These measurements are available in the papers mentioned above.
Timing and spatial scale	Does not apply.
Data exclusions	Does not apply.
Reproducibility	Does not apply.
Randomization	Does not apply.
Blinding	Does not apply.

Did the study involve field work? ☐ Yes ☒ No

Reporting for specific materials, systems and methods

We require information from authors about some types of materials, experimental systems and methods used in many studies. Here, indicate whether each material, system or method listed is relevant to your study. If you are not sure if a list item applies to your research, read the appropriate section before selecting a response.

Materials & experimental systems

n/a	Involved in the study
n/a	☐ Antibodies
n/a	☐ Eukaryotic cell lines
	☒ Palaeontology and archaeology
n/a	☐ Animals and other organisms
n/a	☐ Human research participants
n/a	☐ Clinical data
n/a	☐ Dual use research of concern

Methods

n/a	Involved in the study
	☐ ChIP-seq
	☐ Flow cytometry
	☐ MRI-based neuroimaging

Antibodies

Antibodies used	Does not apply.
Validation	Does not apply.

Eukaryotic cell lines

Policy information about cell lines

Cell line source(s)	Does not apply.

Authentication	Does not apply.
Mycoplasma contamination	Does not apply.
Commonly misidentified lines (See ICLAC register)	Does not apply.

Palaeontology and Archaeology

Specimen provenance	Bear Gulch, USA, published by Whalen & Landman (2022).
Specimen deposition	Royal Ontario Museum (Toronto, Canada) and at the American Museum of Natural History (New York, USA)
Dating methods	Biostratigraphy

☒ Tick this box to confirm that the raw and calibrated dates are available in the paper or in Supplementary Information.

Ethics oversight	Does not apply.

Note that full information on the approval of the study protocol must also be provided in the manuscript.

Animals and other organisms

Policy information about studies involving animals; ARRIVE guidelines recommended for reporting animal research

Laboratory animals	Does not apply.
Wild animals	Does not apply.
Field-collected samples	Only the published materials listed above.
Ethics oversight	Does not apply.

Note that full information on the approval of the study protocol must also be provided in the manuscript.

Human research participants

Policy information about studies involving human research participants

Population characteristics	Does not apply.
Recruitment	Does not apply.
Ethics oversight	Does not apply.

Note that full information on the approval of the study protocol must also be provided in the manuscript.

Clinical data

Policy information about clinical studies

All manuscripts should comply with the ICMJE guidelines for publication of clinical research and a completed CONSORT checklist must be included with all submissions.

Clinical trial registration	Does not apply.
Study protocol	Does not apply.
Data collection	Does not apply.
Outcomes	Does not apply.

Dual use research of concern

Policy information about dual use research of concern

Hazards

Could the accidental, deliberate or reckless misuse of agents or technologies generated in the work, or the application of information presented in the manuscript, pose a threat to:

No	Yes	
☒	☐	Public health
☒	☐	National security
☒	☐	Crops and/or livestock
☒	☐	Ecosystems
☒	☐	Any other significant area

Experiments of concern

Does the work involve any of these experiments of concern:

No	Yes	
☒	☐	Demonstrate how to render a vaccine ineffective
☒	☐	Confer resistance to therapeutically useful antibiotics or antiviral agents
☒	☐	Enhance the virulence of a pathogen or render a nonpathogen virulent
☒	☐	Increase transmissibility of a pathogen
☒	☐	Alter the host range of a pathogen
☒	☐	Enable evasion of diagnostic/detection modalities
☒	☐	Enable the weaponization of a biological agent or toxin
☒	☐	Any other potentially harmful combination of experiments and agents

ChIP-seq

Data deposition

☐ Confirm that both raw and final processed data have been deposited in a public database such as GEO.

☐ Confirm that you have deposited or provided access to graph files (e.g. BED files) for the called peaks.

Data access links<br>*May remain private before publication.*	Does not apply.
Files in database submission	Does not apply.
Genome browser session<br>(e.g. UCSC)	Does not apply.

Methodology

Replicates	Does not apply.
Sequencing depth	Does not apply.
Antibodies	Does not apply.
Peak calling parameters	Does not apply.
Data quality	Does not apply.
Software	Does not apply.

Flow Cytometry

Plots

Confirm that:

☐ The axis labels state the marker and fluorochrome used (e.g. CD4-FITC).

☐ The axis scales are clearly visible. Include numbers along axes only for bottom left plot of group (a 'group' is an analysis of identical markers).

☐ All plots are contour plots with outliers or pseudocolor plots.

☐ A numerical value for number of cells or percentage (with statistics) is provided.

Methodology

Sample preparation	Does not apply.
Instrument	Does not apply.
Software	Does not apply.
Cell population abundance	Does not apply.
Gating strategy	Does not apply.

☐ Tick this box to confirm that a figure exemplifying the gating strategy is provided in the Supplementary Information.

Magnetic resonance imaging

Experimental design

Design type	Does not apply.
Design specifications	Does not apply.
Behavioral performance measures	Does not apply.

Acquisition

Imaging type(s)	Does not apply.
Field strength	Does not apply.
Sequence & imaging parameters	Does not apply.
Area of acquisition	Does not apply.

Diffusion MRI ☐ Used ☒ Not used

Preprocessing

Preprocessing software	Does not apply.
Normalization	Does not apply.
Normalization template	Does not apply.
Noise and artifact removal	Does not apply.
Volume censoring	Does not apply.

Statistical modeling & inference

Model type and settings	Does not apply.
Effect(s) tested	Does not apply.

Specify type of analysis: ☐ Whole brain ☐ ROI-based ☐ Both

Statistic type for inference (See Eklund et al. 2016)	Does not apply.
Correction	Does not apply.

Models & analysis

n/a	Involved in the study
☒	☐ Functional and/or effective connectivity
☒	☐ Graph analysis
☒	☐ Multivariate modeling or predictive analysis

This checklist template is licensed under a Creative Commons Attribution 4.0 International License, which permits use, sharing, adaptation, distribution and reproduction in any medium or format, as long as you give appropriate credit to the original author(s) and the source, provide a link to the Creative Commons license, and indicate if changes were made. The images or other third party material in this article are included in the article's Creative Commons license, unless indicated otherwise in a credit line to the material. If material is not included in the article's Creative Commons license and your intended use is not permitted by statutory regulation or exceeds the permitted use, you will need to obtain permission directly from the copyright holder. To view a copy of this license, visit http://creativecommons.org/licenses/by/4.0/
